# Advances in Knowledge of Candidate Genes Acting at the Beta-Cell Level in the Pathogenesis of T1DM

**DOI:** 10.3389/fendo.2020.00119

**Published:** 2020-03-12

**Authors:** Haipeng Pang, Shuoming Luo, Gan Huang, Ying Xia, Zhiguo Xie, Zhiguang Zhou

**Affiliations:** ^1^Department of Metabolism and Endocrinology, The Second Xiangya Hospital, Central South University, Changsha, China; ^2^Key Laboratory of Diabetes Immunology (Central South University), Ministry of Education, National Clinical Research Center for Metabolic Diseases, Changsha, China

**Keywords:** T1DM, GWAS, pancreatic beta-cell, candidate gene, apoptosis, innate immunity, beta-cell phenotype

## Abstract

T1DM (type 1 diabetes mellitus), which results from the irreversible elimination of beta-cells mediated by autoreactive T cells, is defined as an autoimmune disease. It is widely accepted that T1DM is caused by a combination of genetic and environmental factors, but the precise underlying molecular mechanisms are still unknown. To date, more than 50 genetic risk regions contributing to the pathogenesis of T1DM have been identified by GWAS (genome-wide association studies). Notably, more than 60% of the identified candidate genes are expressed in islets and beta-cells, which makes it plausible that these genes act at the beta-cell level and play a key role in the pathogenesis of T1DM. In this review, we focus on the current status of candidate genes that act at the beta-cell level by regulating the innate immune response and antiviral activity, affecting susceptibility to proapoptotic stimuli and influencing the pancreatic beta-cell phenotype.

## Introduction

The autoimmune disease T1DM (type 1 diabetes mellitus) is characterized by the selective destruction of insulin-producing pancreatic beta-cells by autoreactive T cells, absolute insulin deficiency and subsequent hyperglycemia (1). Both genetic and environmental factors are important in the pathogenesis of T1DM; specifically, environmental factors, such as viral infection and the gut microbiome, may act as triggers that induce the onset of diabetes in individuals with a genetically susceptible background (2–6). However, the precise pathogenic mechanisms have not been established. A more complete understanding of the roles and consequences of risk-associated variants would be beneficial for applying targeted genomic approaches to prevent T1DM.

GWAS (genome-wide association studies) have identified more than 50 genetic risk regions associated with T1DM, but most of these regions comprise several genes, and the risk-conferring variants and genes remain to be defined (7, 8). Of note, more than 60% of these candidate genes are expressed in islets and beta-cells (Table 1), indicating that their roles in the onset and development of T1DM may be at the beta-cell level (13).

**Table 1:** Candidate T1DM genes expressed in islets.

**Candidate gene**	**Region**	**Gene function or potential role in the pathogenesis of T1DM**
BACH2	6q15	Immune response/cytokine-induced apoptosis
BCAR1	16q23.1	
CCR5	3p21.31	Th cell development/chemokine-induced signaling
CCR7	17q21.2	
CD226	18q22.2	Immune regulation
CD69	12p13.31	Signal transduction
CENPW	6p22.32	
CLEC16A	16p13.13	Regulating mitophagy/maintaining beta-cell function
COBL	7p12.1	
CTLA4	2q33.2	T cell activation
CTRB1	16q23.1	
CTSH	15q25.1	Insulin synthesis/cytokine-induced apoptosis
C1QTNF6	22q12.3	BCR signaling pathway/cytotoxicity
DEXI	16p13.13	Regulating the type 1 IFN signaling pathway
ERBB3	12q13.2	Regulating cytokine-induced apoptosis/ modulating APC function
FUT2	19q13.33	Metabolic pathway
GAB3	Xq28	
GLIS3	9p24.2	Maintaining beta-cell mass and function/regulating cytokine-induced apoptosis
GPR183	13q32.3	
GSDMB	17q12	
HIP14	12q14-q12	Apoptosis/insulin production
HLA	6p21.32	Antigen presentation
HORMAD2	22q12.2	
IFIH1	2q24.2	Innate immune response
IKZF1	7p12.2	Immune cell regulation
IKZF3	17q12	Immune cell regulation
ILZF4	12q13.2	
IL2-IL21	4q27	Th cell differentiation/inflammatory response
IL2RA	10p15.1	T cell proliferation
IL7R	5p13.2	Antigen binding/Ig production/cytotoxicity
IL10	1q32.1	Cytokines/inflammatory response
IL-27	16p11.2	Inflammatory response/antiviral effects
INS	11p15.5	Insulin production/positive selection of T cells in the thymus
LMQ7	13q22.2	
ORMDL3	17q12	Protein binding
PRKD2	19q13.32	
PRKCQ	10p15.1	T cell function/apoptosis/innate immune response
PTPN2	18p11.21	Regulating beta-cell apoptosis and insulin secretion
PTPN22	1p13.2	CD4+ T cell activation/autoimmune response
RAC2	22q12.3	
RASGRP1	15q14	Cytokine production/inflammatory response
RNLS	10q23.31	
SH2B3	12q24.12	Growth factor and cytokine signaling
SIRPG	20p13	
SKAP2	7p15.2	
SMARCE1	17q21.2	
STX4	16p12-q11.1	Apoptosis/insulin production
TNFAIP3	6q23.3	Apoptosis/inflammatory response
TYK2	19p13.2	Regulating the type 1 IFN signaling pathway
UBASH3A	21q22.3	Cytokine production/TCR signaling pathway
ZFP36L1	14q24.1	

*Some T1DM candidate genes are expressed in pancreatic islets (9–11). Of note, HIP14 and STX4 are potential candidate genes that were discovered by in silico phenome-interactome network analysis (12)*.

**Figure 1:**
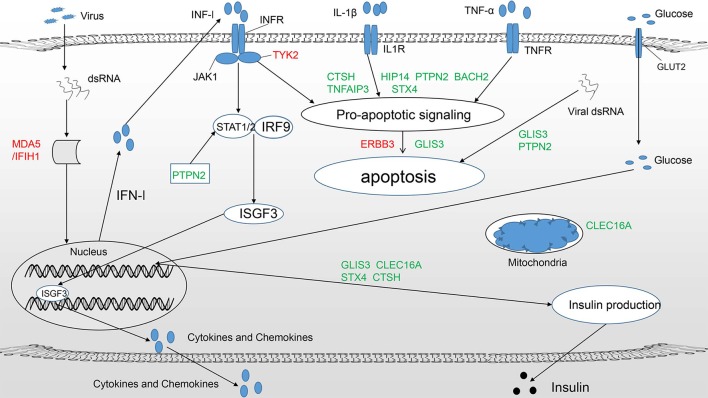
T1DM candidate genes acting at the beta-cell level mainly play a role in three pathways (11): (1) Regulate the innate immune response and pathways important for antiviral activity, such as the type 1 IFN signaling pathway (IFIH1, TYK2, PTPN2). (2) Modulate susceptibility to proapoptotic stimuli (BACH2, TNFAIP3, ERBB3, HIP14, STX4, CTSH, PTPN2). (3) Affect beta-cell phenotypes, primarily insulin production (GLIS3). Candidate genes in green and red represent protective and predisposing candidates, respectively. Some candidate genes clearly participate in more than one pathway.

The results of many studies imply that these candidate genes act at the beta-cell level and contribute to the pathogenesis of T1DM mainly by regulating the innate immune response and antiviral activity, affecting susceptibility to proapoptotic stimuli and influencing pancreatic beta-cell phenotypes (Figure 1) (8, 14). Existing evidence shows that innate immunity is involved in the early induction and amplification of the autoimmune process in pancreatic islets (15, 16). Of all the innate immune responses, the type 1 IFN (interferon) signaling pathway plays a particularly important role in the pathogenesis of T1DM (17). Proinflammatory cytokines and chemokines can suppress beta-cell function, evoke apoptosis and maintain insulitis, which causes the progressive loss of beta-cells (15). Pancreatic beta-cell apoptosis has been viewed as the final and most critical step in the progression of T1DM. If the dying beta-cells are not efficiently eliminated, they become the most significant source of autoantigens, which can worsen insulitis and autoimmunity (18, 19). Beta-cell phenotypes are mainly related to residual function, mass, neogenesis, proliferation and so-called beta-cell suicide (1). Intriguingly, patients with long-standing T1DM have residual insulin-positive beta-cells and exhibit endogenous insulin production (20, 21). Therefore, it will be beneficial to reveal the mechanisms of specific candidate genes that act at the beta-cell level to induce beta-cell dysfunction or death in order to identify new therapeutic targets to treat and cure T1DM.

This review will briefly introduce innate immunity, beta-cell apoptosis and beta-cell phenotypes in patients with T1DM. We focus on the relationship between innate immunity, beta-cell apoptosis, beta-cell phenotypes and T1DM. Later in the review, previous and the most recent findings on T1DM candidate genes acting at the beta-cell level are discussed.

## Innate Immunity and T1DM

As the front line of the immune system, innate immunity plays an important role in eradicating invading pathogens and initiating the adaptive immune response. Humans can detect environmental pathogens through interactions between innate PRRs (pattern recognition receptors), including RLRs (RIG-I-like receptors), TLRs (Toll-like receptors) and NLRs (nucleotide oligomerization domain-like receptors), and PAMPs (pathogen-associated molecular patterns), which are highly conserved structures shared among large groups of microorganisms (22, 23). The recognition of pathogens by PRRs induces a series of innate immune responses, including the production and release of proinflammatory chemokines and cytokines, such as IFNs, IL-1 (interleukin-1), and TNF-α (tumor necrosis factor-α) (22). A moderate innate immune response protects the body against further injury; however, an excessive response can be detrimental in individuals with a predisposing genetic background because of their increased risk of developing autoimmune diseases, such as T1DM (24, 25).

The induction and development of T1DM involve extremely complicated interactions between pancreatic beta-cells and the immune system, which doubtlessly include innate immunity (26). Among all innate immune responses, the type 1 IFN signaling pathway is especially important for beta-cell damage, as demonstrated by pathway analysis (13). A large body of evidence has confirmed the connection between type 1 IFNs and T1DM in both human and animal model studies. It was originally reported that chronic hepatitis patients treated with IFN-α occasionally develop T1DM, indicating a relationship between IFNs and T1DM (27). This finding was further confirmed by the fact that IFN expression levels were elevated in the pancreas of patients recently diagnosed with T1DM (28). Furthermore, self-neutralizing antibodies targeting IFN-α were associated with protection against T1DM in patents with APS1 (autoimmune polyglandular syndrome type 1) (29). It has also been reported that overactivation of the type 1 IFN signaling pathway occurs prior to the appearance of T1DM-associated antibodies, which highlights the role of type 1 IFN as a potentially precipitating factor in the early phase of T1DM (30, 31). These discoveries have been supported by animal experiments in which transgenic mouse models that overexpress IFN-α in beta-cells were shown to develop hypoinsulinemic diabetes, and self-reactive antibodies against IFN-α and its receptors prevented the development of inflammation and diabetes (32, 33).

The mechanisms underlying type 1 IFN-induced T1DM can be divided into two groups (34). In the first group, the non-immunologic mechanisms include the ER (endoplasmic reticulum) stress-mediated impairment of insulin production and the induction of beta-cell apoptosis via the mitochondrial pathway (35, 36). For mechanisms in the second group, type 1 IFN is central to activating innate immunity and adaptive immune responses. The type 1 IFN signaling pathway promotes the production of proinflammatory mediators and the recruitment of innate immune cells, including macrophages, monocytes, NK (natural killer) cells, and DCs (dendritic cells), which can cause and maintain insulitis in a genetically predisposed background (17). Additionally, the IFN-α-triggered overexpression of MHC-| (major histocompatibility complex class I) can evoke more efficient self-antigen presentation and render beta-cells more easily attacked by autoreactive immune cells (35). Under this circumstance, the adaptive immune response is amplified, resulting in the attack of beta-cells by CD8+ T cells.

## Beta-Cell Apoptosis and T1DM

Apoptosis, also termed programmed cell death, is characterized by cell shrinkage, chromatin condensation, DNA and protein cleavage, and the formation of apoptotic bodies accompanied by almost no inflammatory response. This physiological process, which can be divided into the mitochondrial pathway and the death receptor pathway, maintains homeostasis and benefits the organism by eliminating unneeded cells. Both apoptosis pathways function by activating cysteine proteases called caspases.

**Figure 2:**
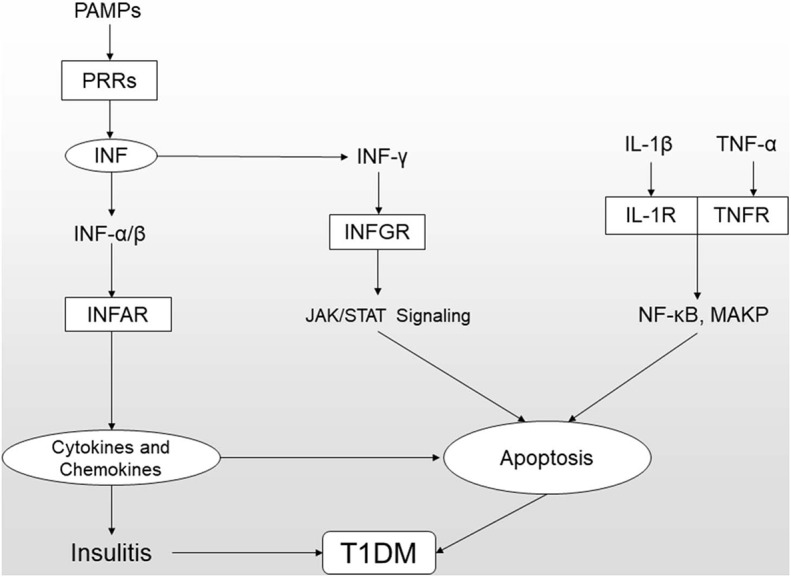
Innate immunity (especially the type 1 IFN signaling pathway) and cytokine-induced apoptosis together contribute to beta-cell death. (1) When PAMPs are bound by PRRs, including the cytosolic receptors RIG-1 and MDA5 and endosomal TLRs, the interactions can promote the synthesis and secretion of type 1 IFNs. IFN-α/β bind their receptor IFNAR and induce the production of cytokines and chemokines that can cause and worsen insulitis and apoptosis. (2) The signaling pathways underlying cytokine-induced apoptosis mainly include (i) JAK/STAT signaling induced by IFN-γ binding to its receptor IFNGR and (ii) NF-κB and MAPK signaling induced by IL-β/IL-R and TNF-α/TNFR.

It is widely accepted that the loss of pancreatic beta-cells due to apoptosis is the significant and final step in the pathogenesis of T1DM (37). The process of apoptosis is closely connected to the innate immune response (Figure 2). For example, the enhanced apoptosis of beta-cells and defective apoptotic cell clearance lead to the leakage of cellular content and exposure of autoantigens, which amplify insulitis and autoimmunity; moreover, DNA accumulation from cellular apoptosis can cause excessive type 1 IFN production. In another example, proinflammatory agents produced by leukocytes, such as IL-1β, IFN-γ, TNF-α, and other soluble mediators, can induce beta-cell apoptosis (19, 38). All these cytokines can activate cytosolic signal transduction pathways that regulate the apoptosis of affected beta-cells. For instance, IL-1β and TNF-α function by activating the NF-κB (nuclear factor-κB), and MAPK (mitogen-activated protein kinase) pathways, and IFN-γ mainly exerts activity via the JAK (Janus kinase)-STAT (signal transducers and activators of transcription) pathway.

After activation, NF-κB translocates to the nucleus and regulates iNOS (inducible nitric oxide synthase) gene expression. Previous evidence shows that NF-κB inhibition prevents cytokine-induced beta-cell apoptosis *in vitro* and *in vivo* and exerts a protective effect against diabetes induced by multiple low-dose treatments with streptozotocin in mice (39–41). After phosphorylation by JAK2, activated STAT1 translocates to the nucleus and regulates the expression of many genes. STAT1 deletion prevents cytokine-induced beta-cell death and diabetes induced by multiple low-dose treatments with streptozotocin in mice (42–44), and STAT1 can also regulate caspase expression (45). The MAPK family includes ERK (extracellular signal-regulated kinase), p38 and JNK (c-Jun N-terminal kinase). The downstream protein kinases and transcription factors, including ATF-2 (activating transcription factor 2), AP-1 (activator protein 1), and c-Jun, modify nuclear gene expression, and AP-1 may be the major transcription factor promoting MAPK-associated apoptosis (46, 47). Additionally, cytokine-mediated signal transduction pathways can interact with each other through MAPKs (47).

## Beta-Cell Phenotypes in T1DM

The beta-cell phenotypes of patients with T1DM mainly relate to beta-cell suicide and the function and mass of residual beta-cells (1). Beta-cell suicide is a consequence of MHC-| overexpression and increased ER stress (1). Overexpression of MHC-| can render insulin-producing beta-cells more sensitive to attack by cytotoxic T lymphocytes, and ER stress is associated with alterations in mRNA splicing and the production of abnormal proteins, which may serve as immunogenic antigens (9, 48). As T1DM develops, most pancreatic beta-cells are lost. However, some studies have identified a substantial number of residual beta-cells in patients with T1DM at diagnosis (8). Furthermore, patients with long-standing T1DM, even more than 50 years, retain identifiable residual beta-cells (21, 49). Moreover, in non-diabetogenic *in vivo* culture, impaired islets from T1DM patients can regain the ability to secrete insulin (50). All these findings demonstrate that the loss of beta-cell function results from both decreased beta-cell mass and decreased function. These studies provide insight into the development of new therapeutic interventions aimed at preserving and augmenting residual beta-cells.

## Candidate Genes in T1DM

To date, more than 50 candidate regions associated with T1DM have been identified by GWAS (7). Some candidate genes are potentially involved in other inflammatory and autoimmune diseases in addition to T1DM, suggesting that they could be key regulators of abnormal autoimmune responses. Previous studies have focused primarily on genes affecting the immune system, such as HLA, CTCL4, and PTPN22. However, gene function at the beta-cell level is receiving increasing attention (Table 1). Genes with such activity exert influence by regulating the innate immune response and antiviral activity (IFIH1, TYK2), influencing susceptibility to proapoptotic stimuli (HIP14, BACH2), and affecting the beta-cell phenotype (GLIS3) (Figure 1). Of note, some genes play a role in both the immune system and at the beta-cell level, such as HLA, INS, and BACH2, which implies that interactions between the abnormal immune system and pancreatic islet beta-cells contribute to the development of T1DM.

### IFIH1

IFIH1 (interferon induced helicase C domain 1), which is located on human chromosome 2q24.2, was identified by GWAS as a candidate gene conferring risk to T1DM; it is expressed in human pancreatic beta-cells and encodes MDA (melanoma differentiation-associated protein 5), a cytoplasmic sensor that recognizes dsRNA, a byproduct of viral replication (51, 52). The interaction between MDA and dsRNA leads to a cascade of antiviral responses, such as the synthesis and secretion of type 1 IFNs (53). IFIH1 promotes cytokine and chemokine production when induced by enterovirus infection or PIC (polyinosinic-polycytidylic acid) (54, 55). Knockout of MDA5 in INS-1E cells and primary beta-cells decreases PIC-induced cytokines and chemokines, which indicates that IFIH1 modulates the local release of inflammatory mediators at the pancreatic beta-cell level (54). Moreover, in NOD mice, partial loss of MDA (MDA+/−) reduces the incidence of spontaneous diabetes, and complete MDA5 deficiency (MDA−/−) fully protects against spontaneous diabetes compared with wild-type (WT) MDA5 status (MDA+/+) (51). Various SNPs (single nucleotide polymorphisms) have been found to confer either susceptibility to or protection against T1DM (56). Among all these mutations, the gain-of-function missense mutation A946T in IFIH1 (rs1990760) has been confirmed to be associated with T1DM as well as several other autoimmune diseases in several independent studies (57–59). IFIH1 A946T confers increased basal and ligand-triggered type 1 IFN expression, and transgenic mice with the A946T risk allele exhibit increased basal type 1 IFN expression (60). Additionally, several studies showed that the IFIH1 A946T risk allele exerts its effect via the IFN-β-mediated response rather than through IFN-α, and IFN-β can promote persistent LCMV (lymphocytic choriomeningitis virus) infection, which causes enduring beta-cell damage (61–65). Intriguingly, a previous study indicated an association between the SNP rs1990760 and seasonal variation in the onset of T1DM and found that the predisposing gene was more likely to be associated with the onset of T1DM in summer (66). This finding may be explained by the theory that T1DM is caused by environmental factors in individuals with a genetically susceptible background. In contrast, two rare protective loss-of-function mutations in IFIH1, rs35667974 (I923V) and rs35744605 (E627X), are associated with potent inhibition of PIC-stimulated IFN-β production (67). In summary, IFIH1 may play an important role in the pathogenesis of T1DM by regulating the innate immune response, especially the type 1 IFN signaling pathway; as the downregulation of IFIH1 may have a positive effect on preventing the onset of T1DM in the initial phase, it may become a useful strategy for preventing T1DM in the future.

### TYK2

Located on human chromosome 19p13.2, TYK2 (tyrosine kinase 2) is a T1DM-associated candidate gene encoding a tyrosine kinase belonging to the JAK family that interacts with the cytoplasmic part of INFAR and plays a role in the type 1 IFN signaling pathway (68). Several SNPs within TYK2 are associated with autoimmune and inflammatory diseases, such as T1DM, RA (rheumatoid arthritis), SLE (systemic lupus erythematosus), MS (multiple sclerosis), and IBD (inflammatory bowel disease) (69–71). A SNP within TYK2 (rs2304256) that causes a missense mutation leading to decreased function has been associated with a decreased risk of developing T1DM (71). Human beta-cells with TYK2 knockout display lower PIC-induced JAK-STAT pathway activation; lower IFN-α, CXCL10, and MHC-| expression; and greater prevention of PIC-induced apoptosis (72). However, mice with lower expression of TYK2, caused by either TYK2 gene knockout or the presence of mutants with reduced TYK2 promoter activity, leading to decreased expression, are more sensitive to virus-induced diabetes, accompanied by higher virus titers and type 1 IFN levels, than mice with WT TYK2 (73). These findings demonstrate that WT TYK2 is crucial for maintaining the appropriate activation of the type 1 IFN signaling pathway. Differences in tissues and species may partially account for the opposing outcomes, but the exact mechanism by which the expression level of this gene regulates T1DM susceptibility remains to be explored. Regardless, there is no doubt that TYK2 can alter the inflammatory response toward beta-cells and may be a promising antidiabetic target.

### PTPN2

PTPN2 (protein tyrosine phosphatase, non-receptor type 2), which is located on human chromosome 18p11, is expressed in human islet cells and exerts negative feedback on the JAK-STAT signaling pathway by dephosphorylating JAKs and STATs (8, 74, 75). In addition to the JAK-STAT signaling pathway, ERK, EGFR (epidermal growth factor receptor) and IRβ (insulin receptor β) are also regulated by PTPN2 (76–78). PTPN2 expression can be upregulated by proinflammatory cytokines and PIC, and PTPN2 knockout in INS-1E cells and primary beta-cells exacerbates PIC-induced apoptosis and proinflammatory cytokine production via the upregulation of STATs (54, 74). In another study, PTPN2 knockout in INS-1E cells, primary rat beta-cells and human beta-cells increased apoptosis by activating JNK, Bim (BH3-only protein) and the intrinsic apoptotic pathway. All these findings show that decreased PTPN2 expression sensitizes beta-cells to apoptosis induced by danger signals, and SNPs within PTPN2 that evoke decreased expression or function may increase the risk of T1DM (8). In addition to apoptosis, insulin secretion is also potentially affected by PTPN2; a previous study found that PTPN2 knockout in mice affected beta-cell function and led to decreased insulin secretion (79).

Of note, all three candidate genes mentioned above participate in regulating the type 1 IFN signaling pathway, and a common trait of risk-conferring variants is the promotion of excessive activation of the inflammatory response, leading to an increased risk of T1DM (13). The evidence not only emphasizes the importance of the type 1 IFN signaling pathway in the pathogenesis of T1DM but also provides a potential treatment strategy, namely, moderately downregulating the expression of type 1 IFNs by using targeted genomic approaches.

### BACH2

Located on human chromosome 6q15, BACH2 (BTB and CNC homology 1, basic leucine zipper transcription factor 2) was traditionally thought to function at the immune system level but has been shown to be expressed in pancreatic beta-cells as well and to be upregulated by proinflammatory cytokines (80). BACH2 knockout in human and mouse beta-cells increases cytokine-induced beta-cell apoptosis via the upregulation of JUN1, BIM and the intrinsic apoptotic pathway; in contrast, BACH2 overexpression has a protective effect on beta-cell apoptosis (80). Moreover, inhibition of BACH2 downregulates PTPN2 expression (80). Although the exact mechanism is still unknown, this finding supports the hypothesis that the network formed by T1DM candidate risk genes renders beta-cells hyper-responsive to danger signals. A recent study found that a BACH2 risk allele (rs3757247) might contribute to the development of insulin-triggered T1DM by affecting the immune response (81). The finding that the BACH2 gene functions at both the immune system and beta-cell levels suggests interplay between these two systems and implies an intricate network underlying T1DM pathogenesis.

### TNFAIP3

TNFAIP3 (TNF-induced protein 3), which is located on human chromosome 6q23, has been identified by GWAS as a candidate gene associated with the onset and pathogenesis of T1DM and other autoimmune diseases, such as RA, IBD and psoriasis (82, 83). The TNFAIP3 gene encodes the zinc finger protein A20, a cytoplasmic ubiquitin-editing protein that is upregulated by cytokines in INS-1E cells and primary mouse islets (83). TNFAIP3 knockout increases INS-1E cell apoptosis induced by proinflammatory cytokines; in contrast, overexpression of this gene decreases apoptosis (84). A20 exerts function via multiple pathways: it negatively regulates NF-κB activation and NO production, inhibits JNK activation, upregulates Akt (a protein controlling beta-cell survival) and subsequently downregulates the intrinsic apoptotic pathway (84). These functions highlight the multiple antiapoptotic effects of A20 in beta-cells (8). In addition to influencing apoptosis, TNFAIP3 also affects beta-cell function by regulating the expression level of ZnT8, which is essential for insulin production and secretion, as determined by experiments showing that TNFAIP3 overexpression protects ZnT8 from cytokine-induced downregulation (85). Furthermore, a SNP in the non-coding region of TNFAIP3 (rs2327832) is associated with lower C-peptide and higher HbA1c (hemoglobin A1c) levels, which indicates reduced beta-cell function and impaired glycemic control in children with recent onset of T1DM (84). Although further investigation in different cohorts is needed, this finding provides evidence that A20 influences beta-cell death and function. Another recent study indicated that islet allografts with A20 upregulation show increased survival via NF-κB inhibition, AP-1 reporter activation and CXCL10 transcription (86), which sheds some light on the possibility of reducing immunosuppression therapies after islet transplantation and increasing the success rate of this operation.

### ERBB3

Located on human chromosome 12q13.2, the ERBB3 (erb-b2 receptor tyrosine kinase 3) gene is known for its role in cancer. The ERBB3 gene encodes a protein in the EGFR family that functions as a heterodimer with other EGFR family members (87). The SNP rs2292239, located in intron 7 of ERBB3, is associated with T1DM, residual beta-cell function and metabolic control (88–90). Previous studies focused on this gene reported that it confers a risk for T1DM by modulating APC function to exert immunoregulatory effects (91). A later study demonstrated that ERBB3 also affects beta-cell apoptosis (89). ERBB3 knockdown decreases basal and cytokine-induced apoptosis, but ERBB3 expression is downregulated by proinflammatory cytokines, indicating that this gene may participate in negative regulation by cytokines (89). Thus, further investigation is needed to resolve the contradiction that the ERBB3 gene is downregulated by proinflammatory cytokines but increases cytokine-induced apoptosis. Additionally, this contradiction may suggest that additional unknown mechanisms affect beta-cell death.

### HIP14

The HIP14 (huntingtin-interacting protein 14) gene located on human chromosome 12 encodes a palmitoyl transferase that is highly expressed in the brain (92). HIP14 was identified as a T1DM candidate protein by *in silico* phenome-interactome network analysis (12). HIP14 is expressed in pancreatic islets, with predominant expression in beta-cells (12). Unlike PTPN2, BACH2, and TNFAIP3, which are upregulated by proinflammatory cytokines, HIP14 is downregulated by cytokines (12). HIP14 is thought to participate in T1DM development through interactions with two proteins physically associated with T1DM, HTT (huntingtin protein), and GAD65 (glutamate decarboxylase 65) (12). HIP14 knockout leads to increased apoptosis, whereas HIP14 overexpression results in decreased apoptosis due to reduced NF-κB activation (12). Another study indicated that caspase 6, which plays an important role in apoptosis, can be inhibited by the palmitoyl transferase activity of HIP14 in the mouse brain (93). However, further investigation is needed to clarify whether this effect also occurs in pancreatic beta-cells. In addition to apoptosis, insulin release is also affected by the palmitoyl transferase activity of HIP14 (12). The knockout of HIP14 and overexpression of mutant HIP14 lacking the palmitoyl transferase domain lead to decreased insulin release, indicating that the palmitoyl transferase activity of HIP14 participates in insulin secretion (12). In summary, HIP14 may contribute to the development of T1DM by regulating beta-cell apoptosis and insulin secretion, but more evidence is required to determine whether it is a candidate risk gene of T1DM.

### STX4

Located on human chromosome 16, the STX4 (syntaxin 4) gene is situated within the T1DM susceptibility region, and similar to HIP14, the Stx4 protein encoded by STX4 was identified as a T1DM candidate protein by *in silico* phenome-interactome network analysis (12, 94). STX4, which localizes to the plasma membrane, is associated with insulin secretion (94). STX4 overexpression restricted to pancreatic beta-cells increases the capacity for insulin secretion, promotes glucose tolerance and protects STZ-treated mice from developing diabetes (94). Furthermore, increased STX4 expression can downregulate the expression of chemokine genes associated with inflammation and the apoptosis of pancreatic islets, such as CXCL9, CXCL10, and CXCL11 (94). Additionally, increased STX4 expression leads to decreased apoptosis by decreasing the translocation and activation of NF-κB (94). In conclusion, STX4 can influence both insulin secretion and beta-cell apoptosis, and it may be a novel target for the treatment of T1DM.

### CLEC16A

CLEC16A (c-type lectin domain family 16, member A), which is located on human chromosome 16p13, encodes a membrane-associated endosomal protein that has been associated with T1DM, MS, primary adrenal insufficiency and other inflammatory and autoimmune diseases (95–97). CLEC16A plays a role in mitochondrial autophagy (mitophagy), a process to eliminate unhealthy mitochondria that is essential for maintaining beta-cell function, glucose homeostasis and GSIS (glucose-stimulated insulin secretion); inhibition of the CLEC16A-related pathway impairs beta-cell oxygen consumption and insulin secretion (98). A ubiquitin-dependent tripartite composed of CLEC16A, NRDP1, and USP18 was reported to act as a regulator of beta-cell mitophagy (99). A previous study found that pancreas-specific CLEC16A deficiency led to impaired glucose tolerance, ER stress and GSIS in mice, and a SNP in the CLEC16A gene (rs12708716) associated with reduced expression resulted in impaired beta-cell function in humans (98). A recent finding indicated that risk variants within CLEC16A might lead to insulin-triggered T1DM due to less efficient negative selection in the thymus (81). These findings shed light on how mitophagy maintains and promotes beta-cell function and suggest the candidate gene CLEC16A as a new potential therapeutic target for T1DM.

### DEXI

DEXI, which is located in the same region as the CLEC16A gene, encodes a dexamethasone-induced protein of unknown function that is highly expressed in human pancreatic islets; this gene has been implicated in the pathogenesis of T1DM and other autoimmune diseases, such as MS (95, 100). According to gene expression analysis, SNPs within the CLEC16A gene modulate the expression level of DEXI, suggesting DEXI as a potential candidate gene related to T1DM (101). A previous study found that DEXI knockout led to the decreased activation of STAT1 and production of proinflammatory chemokines, such as CCL5, CXCL9, and CXCL1, in PIC-treated INS-1E cells, and DEXI overexpression has the opposite results (100); moreover, DEXI was shown to modulate IFN-β transcription (100). Based on these findings, the researchers concluded that DEXI might participate in the pathogenesis of T1DM by regulating the type 1 IFN signaling pathway (100). However, another recent study found that DEXI knockout did not alter the frequency of diabetes or influence the protective effect afforded by CLEC16A knockout in NOD mice (102). These researchers concluded that CLEC16A, rather than DEXI, is a causal gene of T1DM within human chromosome region 16p13 (102). Different cell types and species may partly explain the opposite conclusions, but the precise underlying mechanisms remain to be further investigated.

### CTSH

Located on human chromosome 15q25.1, the CTSH (cathepsin H) gene encodes a lysosomal cysteine protease that is expressed in human pancreatic islet beta-cells and downregulated upon exposure to proinflammatory cytokines. Overexpression of CTSH leads to decreased cytokine-induced apoptosis by decreasing the activation of the JNK1/2 and p38 pathways and the production of proapoptotic factors, including c-Myc, Bim and DP5 (death protein 5), in insulin-producing INS-1 cells (103). In addition to its effects on beta-cell apoptosis, CTSH overexpression also resulted in increased insulin accumulation in the medium and higher Ins2 levels. In line with this finding, CTSH(-/-) mice have lower plasma insulin levels than WT mice (103). These facts indicate the antiapoptotic effects of CTSH and its ability to enhance beta-cell function. A SNP in CTSH (rs3825932) associated with lower expression affects disease progression in children with newly diagnosed T1DM in an allele dose-dependent manner, characterized by the requirement for a higher daily insulin dose and a lower chance of remission (103). This variant also influences beta-cell function in healthy adults (103). However, another SNP (rs2289702) in low LD (linkage disequilibrium) with rs3825932 was recently discovered to have an adverse effect; rs2289702 correlates with decreased CTSH expression and plays a protective role in T1DM (104). The researchers speculated that increased CTSH expression might lead to an excessive innate immune response, thus increasing the risk of T1DM, based on the fact that CTSH can increase the activation of TLR3, a protein expressed in human islets, via cleavage of the N-terminus (104). Further investigation is needed to clarify whether this gene has protective properties and to elucidate its underlying mechanisms.

### GLIS3

GLIS3 (Gli-similar 3), which is located on human chromosome 9p24.2, encodes a transcription factor in the zinc finger family; this gene was identified by GWAS as a candidate gene for both T1DM and T2DM (82, 105). It plays an important role in the development and generation of beta-cells by maintaining mature beta-cell mass and function and INS gene expression (106, 107). Loss-of-function mutations within GLIS3 lead to a rare syndrome mainly characterized by neonatal diabetes and congenital hypothyroidism in humans, and in accordance with this, GLIS3(−/−) mice develop neonatal diabetes caused by impaired pancreatic beta-cell generation and insulin production (108, 109). Additionally, GLIS3 knockout increases basal and proinflammatory cytokine-induced apoptosis by promoting the formation of a proapoptotic splice variant of BIM (110). These findings indicate that GLIS3 protects against T1DM by maintaining beta-cell function and mass and by exerting antiapoptotic effects. It is conceivable that we can prevent the onset of T1DM by enhancing GLIS3 in the future.

## Discussion

As evident from the above discussion, candidate genes acting at the beta-cell level play important roles in the onset and development of T1DM and, together with genes acting at the immune system, constitute the complete pathogenic network. There is a critical need to elucidate the exact underlying mechanisms of these genes to fully understand T1DM. Additionally, advances in this field will provide new therapeutic strategies for T1DM, i.e., avenues to moderately downregulate the innate immune response and cytokine-induced apoptosis and to strengthen residual beta-cell function and viability by using genetic engineering techniques.

T1DM is a multifactorial autoimmune disease, and its precise mechanisms are still unknown. However, it is widely accepted that a combination of environmental and genetic factors contributes to the onset and pathogenesis of T1DM. Candidate genes identified by GWAS influence not only the immune system but also pancreatic islet beta-cells. Some studies have revealed that risk genes act at the beta-cell level mainly through modulating the innate immune system, antiviral activity, and beta-cell apoptosis and phenotypes, and understanding potential pathogenic mechanisms will be helpful in the development of new treatments. However, T1DM is an extremely complex and heterogeneous disease, and these characteristics may be attributed to different genetic backgrounds and environmental components. To develop a more precise predictive model and more effective treatment and prevention measures, it is necessary to fully elucidate the pathogenic network of T1DM. A scoring system for quantifying the genetic and environmental elements may help considerably. To reach this ambitious goal, we propose roughly dividing the process into the following steps. First, screen candidate risk genes for T1DM and establish a pathogenic network comprising genetic and environmental elements. Next, assign these elements a value according to importance in conferring risk for T1DM and build a formula based on epidemiological information. Finally, assess the susceptibility of developing T1DM using the novel formula, and take individualized prevention measures in the predisposed population. Since the pathogenic mechanisms are not fully understood, there is still a long way to go to achieve this goal.

## Author Contributions

HP searched references, wrote the first draft of the paper and revised the text. SL, GH, and YX critically revised the text and provided substantial scientific contributions. ZZ and ZX proposed the project and revised the manuscript. All the authors approved the final version of the manuscript.

### Conflict of Interest

The authors declare that the research was conducted in the absence of any commercial or financial relationships that could be construed as a potential conflict of interest.
